# Risk factors for gestational diabetes mellitus in a sample of pregnant women diagnosed with the disease

**DOI:** 10.1186/1758-5996-7-S1-A80

**Published:** 2015-11-11

**Authors:** Renata Selbach Pons, Fernanda Camboim Rockett, Bibiana de Almeida Rubin, Maria Lúcia Rocha Oppermann, Vera Lúcia Bosa

**Affiliations:** 1 Universidade Federal do Rio Grande do Sul, Porto Alegre, Brazil

## Background

The known risk factors for Gestational Diabetes Mellitus (GDM) are advanced age (≥35 yrs.), overweight or obesity, excessive gestational weight gain, excessive central body fat deposition, family history of diabetes, short stature (<1.50 m), excessive fetal growth, polyhydramnios, hypertension or preeclampsia in the current pregnancy, history of recurrent miscarriage, offspring malformation, fetal or neonatal death, macrosomia, GDM during prior pregnancies and polycystic ovary syndrome. In addition to the most common factors the sedentary lifestyle may also be a risk factor for GDM.

## Objective

To identify the presence of GDM risk factors during pregnancy at the time of diagnosis of this pathology.

## Materials and methods

Cross-sectional study of 76 pregnant women who were referred to a multidisciplinary clinic for high risk pregnancies, in a tertiary hospital in southern Brazil, at the time of GDM diagnosis. A trained interviewer administered a questionnaire to gather sociodemographic, clinical, anthropometric and lifestyle habits data. Pre-pregnancy nutritional status and weight gain were classified according to the Institute of Medicine guidelines. Current Body Mass Index (BMI) was classified by gestational week, according to Atalah. Because the risk of developing GDM gradually increases in overweight and obese women, the total sample was divided into three pre-pregnancy BMI groups for comparison: BMI <25 kg/m^2^, BMI ≥25 kg/m^2^ and <30 kg/m^2^ and BMI ≥30 kg/m^2^. Pearson Chi-square test, analysis of variance and Kruskal-Wallis were employed.

## Results

**Figure 1:**
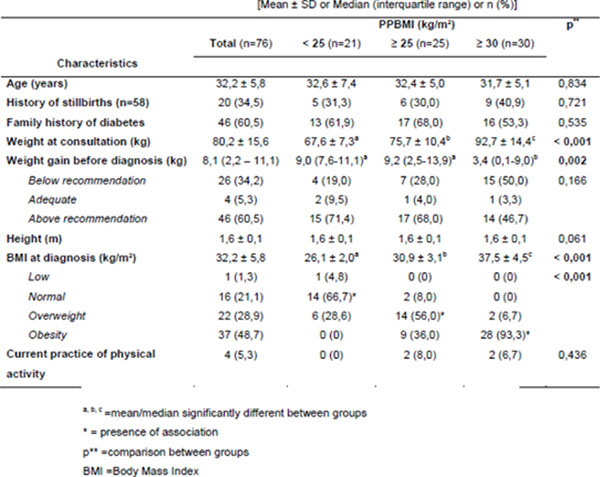
Risk factors related to the development of Gestational Diabetes Mellitus stratified by pre-pregnancy Body Mass Index (PPBMI) classification in a sample of pregnant women at diagnosis of the disease, Rio Grande do Sul/Brazil, 2014.

Seventy-six women were evaluated, 53 (69.7%) had 10 yrs. of education or less and 23 (30.3%) had 11 yrs. or more. In this sample there was a high prevalence of: unfavorable pre-pregnancy nutritional status (overweight or obesity), gestational weight gain above recommendations (PPBMI <25 kg/m^2^ is associated with greater weight gain compared to obese), family history of diabetes, advanced age (38.2%) and low levels of physical activity (data shown in Figure 1).

## Conclusion

We observed a high prevalence of risk factors for GDM in this sample. An adequate pre-pregnancy nutritional status was associated with gestational weight gain above recommendations. These Results emphasize the need for pregnant women to be professionally monitored so that modifiable risk factors can be managed.

